# Middle Adulthood Relationships and Later Cognitive Function: An 18-Year Study of Mexican-Origin Adults

**DOI:** 10.1093/geroni/igaf122.4163

**Published:** 2025-12-31

**Authors:** Ariana Guenther, Olivia Atherton, Angelina R Sutin, Richard Robins, Tomiko Yoneda

**Affiliations:** University of California, Davis, Davis, California, United States; University of California, Riverside, Riverside, California, United States; Florida State University College of Medicine, Tallahassee, Florida, United States; University of California, Davis, Davis, California, United States; University of California, Riverside, Riverside, California, United States

## Abstract

Extensive evidence shows that close relationships matter for cognitive health, serving as both risk and protective factors. However, most existing literature focuses on older White adults, relies on self-reports, and centers on spousal relationships. Less is known about whether midlife spousal and child relationship experiences affect cognitive function over time, particularly in underrepresented communities. Our study addresses these gaps by focusing on middle adulthood, a period when people balance caregiving, work, and evolving family roles. Specifically, we ask whether relationship quality with partner and child (assessed via parent and child reports) shapes initial levels and long-term change in cognitive function. Drawing on data from an 18-year longitudinal study of Mexican-origin adults (N = 1,111; Mage = 38.3 at baseline), we used latent growth curve modeling to examine several relationship factors (aggregated across Years 1-10) as predictors of level and change in cognitive function assessed at Years 12, 16, and 18. Findings showed that higher relationship stability, satisfaction, warmth, and social support were associated with higher baseline cognitive function. However, higher satisfaction, warmth, and social support were also associated with steeper cognitive declines over time. In contrast, relationship instability, hostility, and conflict were unrelated to cognition, except for relationship instability predicting faster decline. Higher parent–child conflict also predicted higher baseline cognition but steeper decline. Together, these findings emphasize complex links between relationship factors and cognitive aging, suggesting supportive relationships are protective for cognition early in middle adulthood, but may also be associated with more decline later in midlife.

